# Dysautonomia in Alzheimer’s Disease: A Systematic Review

**DOI:** 10.3390/brainsci15050502

**Published:** 2025-05-14

**Authors:** Marianna Papadopoulou, Maria-Ioanna Stefanou, Eleni Bakola, Christos Moschovos, Athanasia Athanasaki, Evdoxia Tsigkaropoulou, Ioannis Michopoulos, George P. Paraskevas, Rossetos Gournellis, Georgios Tsivgoulis

**Affiliations:** 1 Department of Physiotherapy, University of West Attica, 12243 Athens, Greece; 2 Second Department of Neurology, Attikon University Hospital, School of Medicine, National and Kapodistrian University of Athens, 12462 Athens, Greece; marianna421@hotmail.co.uk (M.-I.S.); elbakola@yahoo.gr (E.B.); moship@windowslive.com (C.M.); athanasia.athan@yahoo.gr (A.A.); geoprskvs44@gmail.com (G.P.P.); tsivgoulisgiorg@yahoo.gr (G.T.); 3 First Department of Psychiatry, Eginition University Hospital, School of Medicine, National and Kapodistrian University of Athens, 11528 Athens, Greece; evitsigaropoulou@yahoo.com; 4 Second Department of Psychiatry, Attikon University Hospital, School of Medicine, National and Kapodistrian University of Athens, 12462 Athens, Greece; yanmih@yahoo.com (I.M.); rossetosgournellis@gmail.com (R.G.)

**Keywords:** Alzheimer’s disease, dysautonomia, heart rate variability, orthostatic hypotension, sympathetic skin response

## Abstract

**Background**: Alzheimer’s disease (AD) is the most common cause of dementia. In addition to cognitive decline, non-cognitive symptoms, including dysautonomia, have been reported, although these symptoms are rarely acknowledged by patients. Dysautonomia in AD is thought to arise from either cholinergic deficits or hypothalamic involvement. A wide range of tests has been used to investigate the role of the autonomic nervous system; however, the results have been inconsistent. **Aim**: To systematically review all published research investigating autonomic nervous system (ANS) involvement in patients with AD. A comprehensive literature search was conducted in December 2024 across the following databases: PubMed, Cochrane Library, ScienceDirect, and Scopus. **Results**: A total of 1422 records were identified, of which 30 studies fulfilled the inclusion criteria and were included in the review. Several autonomic tests were employed, with Heart Rate Variability (HRV) being the most frequently used. Other tests included assessments of orthostatic hypotension (OH), postprandial hypotension (PPH), sympathetic skin response (SSR), the tilt test, 123I-MIBG cardiac scintigraphy, norepinephrine (NE) measurements in serum and cerebrospinal fluid, and baroreflex sensitivity. In most studies, AD patients were compared to either healthy controls or patients with other types of dementia. **Discussion**: The primary finding of this review is that, although patients with AD rarely report dysautonomic symptoms, they frequently exhibit abnormal results on various autonomic tests. In some cases, these findings were sufficient to differentiate AD patients from healthy controls as well as from patients with Diffuse Lewy Body disease (DLB). The inconsistency in reporting symptoms, along with the variability in test results, suggests that autonomic dysfunction in AD may be under-recognized and warrants further investigation. **Conclusions**: The heterogeneity of the included studies limits the generalizability of the results. However, given the potential impact of dysautonomia on both quality of life and mortality, it is recommended that AD patients be systematically assessed for autonomic dysfunction. Even in the absence of overt symptoms, appropriate treatment should be considered where indicated to mitigate potential risks.

## 1. Introduction

Dementia is a leading cause of disability and dependency globally [1]. According to the American Psychiatric Association’s Diagnostic and Statistical Manual (DSM-5), dementia is diagnosed when there is cognitive decline from a previous higher level that has become severe enough to interfere with social and/or occupational functioning [2]. The diagnosis requires decline in at least two cognitive domains, one of which must be memory. Alzheimer’s disease (AD) is the most common cause of dementia and is characterized by the intercellular accumulation of the protein fragment beta-amyloid and the abnormal extracellular accumulation of the protein tau, which leads to neuronal death [3]. This neurodegenerative process results in brain atrophy.

The incidence of both dementia and AD increases dramatically with age, doubling approximately every five years after age 65 [4]. Symptoms of cognitive decline evolve over the course of several years. In this disease continuum, the clinical spectrum ranges from an asymptomatic phase—when patients exhibit only brain changes—to more severe stages, in which patients require assistance with all daily activities.

Although AD is primarily considered a memory and cognitive disorder—due to the predominant involvement of the hippocampus and neocortex—non-cognitive symptoms are also reported and are attributed to the spread of AD pathology, such as amyloid plaques and neurofibrillary tangles in other brain regions [5]. Deposits of beta-amyloid are often found in various locations in elderly individuals, including the anterior horns of the cervical spinal cord, whereas tangles have been observed in nearly all organs [6]. The most commonly reported non-cognitive symptoms are psychiatric symptoms, including depression, apathy, anxiety, agitation, disinhibition, hallucinations, and delusions [6]. Many theories have been proposed to link AD and mental disorders, especially depression, but none are fully satisfactory or widely accepted. These theories range from considering depression as a psychological reaction [7] or as a compensatory mechanism [8] to the more complex theory involving several neuronal circuits with a common pathophysiology, encompassing neuroinflammation and hypometabolism, common to both disorders [9,10].

Other, less common non-cognitive symptoms include alterations in body weight, sleep-wake disorders, and neuroendocrine changes, all of which are attributed to hypothalamic involvement [5], as confirmed by the findings of plaques and tangles in the area [11], as well as gait and balance dysfunction, olfactory dysfunction, and reduced pain prevalence [6].

Autonomic dysfunction is also observed in various dementia syndromes, AD being one of them. This is supported by the fact that cardiovascular factors, such as hypertension, are considered risk factors for developing AD [12,13]. A common pathway between cardiovascular dysfunction and cognitive decline is the cholinergic system. The cholinergic system is predominantly and early affected in AD, leading to a reduction in cerebral blood flow as a result of the loss of cortical perivascular cholinergic nerve terminals [14,15]. Furthermore, several brain areas affected in parallel in AD, such as the hypothalamus, locus coeruleus, insular cortex, and brainstem, may alter autonomic function [16].

No disease-specific clinical scale exists to assess clinical dysautonomia in AD. The prevalence of clinical dysautonomia can be evaluated using scales validated in other neurodegenerative diseases, such as Parkinson’s disease (PD) [17], or the Composite Autonomic Symptom Score (COMPASS) [18].

Several tests have been used across studies to investigate dysautonomia in AD. Heart rate variability (HRV) is the most commonly used, since it is non-invasive and does not require cooperation from patients with dementia. Power spectral analysis of HRV provides a quantitative evaluation of the sympathovagal equilibrium [19]. The low-frequency (LF) spectral component (0.04–0.15 Hz) is mediated by the sympathetic branch in the HRV signal, while the high-frequency (HF) spectral component (0.15–0.40 Hz) reflects parasympathetically mediated activity. The very low frequency (VLF) (<0.40 Hz) is related to long-term regulatory mechanisms and implicates both sympathetic and parasympathetic branches. The LF/HF ratio indicates the balance between the sympathetic and parasympathetic systems. Another measurement is the total spectral power (TSP) and the normalized units of LF and HF according to the Task Force guidelines [20]. Normal HRV is regarded as a reliable index of autonomic nervous system (ANS) integrity, as seen in diabetic neuropathy [21]. The association between HRV and cognitive decline has been discussed and is considered amphidromic; impaired HRV may lead to increased blood pressure variability, resulting in cerebral hypoperfusion and, consequently, cognitive impairment. The association between HRV and cognitive decline has been discussed and considered to be amphidromical; impaired HRV may produce increased blood pressure variability, thus leading to cerebral hypoperfusion and consequently to cognitive impairment [22]. On the other hand, the neurodegenerative process in AD may cause dysautonomia and HRV abnormalities [23]. In this respect, an abnormal HRV result in AD patients may be viewed either as a consequence of the disease process or as a risk factor for developing AD.

Similarly, blood pressure variability (BPV) is another tool to investigate patients at risk of developing dementia. It has been shown that high BPV and/or high mean blood pressure are associated with the risk of dementia of any type. Cognitive decline is attributed to cerebral small vessel disease; thus, one can assume that apart from the degenerative process, vascular pathology also contributes to the process preceding dementia [24]. R-R interval variation test (RRIV) is also a simple and reliable test to measure heart rate variability. R-R interval measures the length of a ventricular cardiac cycle between two successive R waves [25].

Tilt testing is another autonomic test that is cheap, safe, and well-tolerated, and can be informative in cases of unexplained drop attacks or when description is atypical. Most studies align with the 2008 Newcastle protocols [26]. A description of the test is found in the Mellingsæter et al. study [27]. Patients remain supine on a footplate support-type tilt table for 5–10 min with continuous electrocardiogram (ECG) and blood pressure monitoring. They are then tilted to 30° for 10 min, followed by tilting back to the supine position for another 10 min before being tilted to 70° for 6–8 min. In the deep-breathing test, six consecutive maximal expirations and inspirations are performed, with continuous ECG and blood pressure monitoring.

Orthostatic hypotension (OH) is the inability to sustain blood pressure after standing and has been linked to dementia [28]. Sustained OH is defined as a fall in systolic blood pressure of greater than 20 mm Hg or diastolic blood pressure of greater than 10 mm Hg, which does not return to baseline within 30 s from the start of the active stand [29]. As in the case of HRV, OH might be a risk factor for dementia or, alternatively, a consequence of it. A drop in postural blood pressure indicates dysregulation of the cholinergic and noradrenergic systems involved in blood pressure regulation. On the other hand, in the course of AD, OH can contribute to frontal brain changes due to lower cerebral blood flow and secondary vascular brain lesions, which may contribute to the pathogenesis of the disease [30].

Postprandial hypotension (PPH) is a decrease in systolic blood pressure of 20 mm Hg or more after meals. PPH occurs in the elderly, particularly in nursing homes, and in age-related brain disorders such as PD and chronic autonomic failure, contributing to dizziness and falls in older adults. The mechanism of postprandial hypotension is not fully understood and may include inadequate sympathetic nervous system compensation, impairment in baroreflex, inadequate cardiac output, or impaired peripheral vasoconstriction [31].

Baroreflex (BR) is activated to modulate heart rate in response to abrupt blood pressure changes. It requires continuous and synchronous measurement of blood pressure and ECG and has also been used in AD to assess dysautonomia. The BR functions as a reflex loop for short-term blood pressure regulation, involving the heart, blood vessels, and cerebrum. It consists of parasympathetic inhibition, resulting in an increase in heart rate, followed by sympathetic activation leading to vasoconstriction [32].

Sympathetic Skin Response (SSR) is a neurophysiological procedure that measures changes in electrodermal activity as a response to a noxious stimulus, most commonly electric shock delivered to a peripheral nerve. Abnormal SSR measurements, such as prolonged latencies and low amplitudes of the evoked potentials, or, in more severe cases, the complete absence of the response, typically result from impairment of efferent small fibers in the peripheral nervous system. SSR has also been used to investigate central nervous system disorders affecting the ANS [33]. A unique aspect of the test is that it investigates solely one branch of ANS, the sympathetic nervous system.

Lastly, 123I-Meta-Iodobenzylguanidine Cardiac Scintigraphy is used to measure the ratio of the average pixel counts in the heart (H) to those in the mediastinum (M) (the H/M ratio) and the washout rate in the heart. It also reflects postganglionic sympathetic activity. Because scintigraphy requires the use of a radioisotope and a long testing period, it is not routinely used in clinical settings [34,35].

Although there is evidence suggesting autonomic dysfunction in AD, these symptoms are rarely reported and discussed. Autonomic dysfunction has important clinical implications, ranging from dizziness and falls to syncope and sudden death, and deserves further study to raise awareness among clinicians treating patients with AD. This systematic review aims to present all published work investigating autonomic dysfunction in AD and discuss the implications for clinical practice.

## 2. Materials and Methods

### 2.1. Review of the Literature

**Figure 1:**
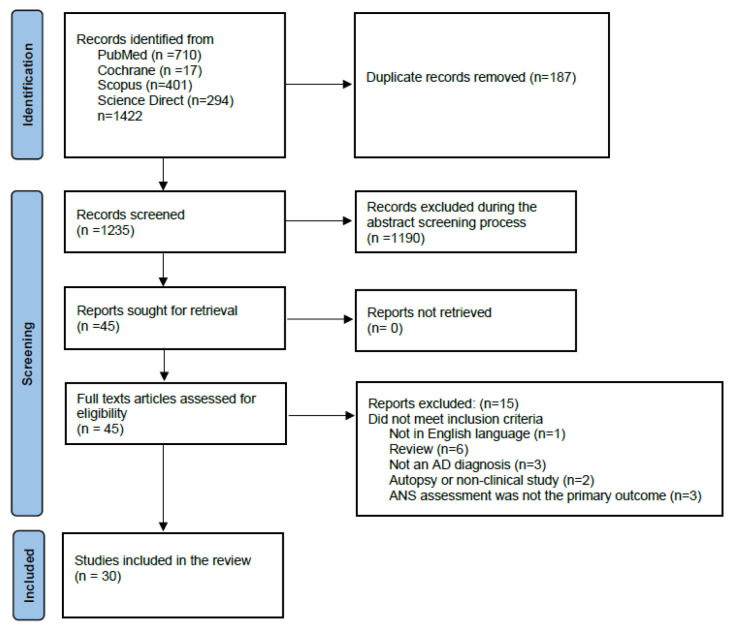
Prisma flow diagram.

This systematic review was conducted according to the PRISMA (Preferred Reporting Items for Systematic Reviews and Meta-Analyses) guidelines [36] Figure 1. The protocol of the present review has been pre-registered with the Open Search Foundation (OSF) (Registration: osf.io/ab987).

A comprehensive literature search was performed to identify eligible studies investigating autonomic dysfunction in AD, published in the PubMed, Cochrane Library, ScienceDirect, and Scopus databases, from the inception of each database to December 23, 2024. No language or other search restrictions were applied. The literature search included both Medical Subject Headings (MeSH) and free-text terms. The following search strategy was implemented across different databases: ‘Alzheimer’s disease’ AND ‘dysautonomia’ OR ‘HRV’ OR ‘SSR’ OR ‘orthostatic hypotension’. The details of the search are given in Supplement File S1.

The reference lists of all the appraised articles were screened for relevant citations that might have been missed in the electronic searches. Only full-text original articles, written in English and published in indexed peer-reviewed journals, were eligible for inclusion. Once all articles were identified, duplicates were removed. Two reviewers (MP and IM) independently screened the titles and abstracts for eligibility and examined the full text of the articles for final decision (Figure 1). In case of disagreement, consensus was reached regarding the inclusion of each study. If needed, a third reviewer was consulted (RG). Excluded studies based on full text search are provided in Supplementary File S2.

### 2.2. Inclusion Criteria

1. All types of observational studies were included: case-control studies, cohort studies, cross-sectional studies, and retrospective studies
2. A diagnosis of Alzheimer’s disease was a prerequisite
3. Studies including patients suffering from dementias other than AD were considered when sufficient data on AD were provided
4. Primary outcome assessment of autonomic dysfunction with specific measurement tools

### 2.3. Exclusion Criteria

1. Reviews (systematic or other) and meta-analyses
2. Animal studies
3. Autopsy studies with no clinical correlation to dysautonomia
4. Studies including patients suffering from diseases that may affect the autonomic nervous system (e.g., diabetes mellitus, amyloidosis etc.)

### 2.4. Data Extraction

Both reviewers extracted data from 30 eligible studies, which included: authors, date of publication, study type, number of subjects, epidemiological data (age, sex), clinical presentation, autonomic evaluation, and outcomes. Data are presented in brief in Table 1.

### 2.5. Risk of Bias

Critical appraisal of the selected cross-sectional studies was conducted using the Joanna Briggs Institute JBI tool [61,62]. It consists of eight questions that assess the clarity of inclusion criteria, the description of subjects and setting, the validity and reliability of the exposure measurement, the objectivity of the criteria used for measurement of the condition, the identification of confounding factors, the strategies used to deal with confounding factors, the validity and reliability of outcome measurement, and the statistical analysis. Each checked question was scored either as Yes, No, Unclear, or Not Applicable. Each study is rated overall as Included or Excluded or Further Information Requested—Supplement File S3.

## 3. Results

### 3.1. Demographic and Clinical Characteristics

A total of 30 studies have been identified and reviewed. The demographic and clinical characteristics are presented in summary in Table 1. The majority of studies were cross-sectional in design. Two were retrospective, one by Haglund et al. and one by Kim et al. [37,40], and two were observational studies without control group by Toledo and Junqueira and by Freidenberg et al. [42,48]. The oldest study dates back to 1984 by Raskind et al. [55] and the most recent to 2024, by Haglund et al. [37].

Most studies compared AD patients to healthy controls (HC) and/or patients suffering from other types of dementia, such as vascular dementia (VAD), mild cognitive impairment (MCI), dementia with Lewy bodies (DLB), or Parkinson’s disease dementia (PDD). The groups were age- and gender-matched. Mini Mental State Examination (MMSE) results, reflecting dementia severity, were reported in sixteen studies, and the Cambridge Cognition Examination (CAMCOG) was reported in three [42,59,60]. Global Deterioration Scale (GDS) was reported in two [54,55], and Frontal Assessment Battery (FAB) was reported in one [34]. In the remaining ten studies, no information regarding the cognitive status of participants was provided. In most studies, patients were excluded if they were receiving medications that affected the ANS or if they were receiving medications that were discontinued prior to study entry. In seven studies [29,34,37,41,52,59,60], this information was not reported. Moreover, in the study by Giubilei et al. [45], the authors reported that when patients were treated with cholinesterase inhibitors, autonomic dysfunction was normalized. In the study by van Beek et al. [49], the authors showed that galantamine, a cholinesterase inhibitor, did not negatively affect blood pressure or heart rhythm. Finally, Meel-van den Abeelen et al. [32] suggested that cholinesterase inhibitors might slow disease progression through their effects on blood pressure and other cardiovascular factors.

Information regarding the clinical phenotype of dysautonomia was even scarcer. In two studies by Allan et al. [43,60], the results of the Ewing’s Battery were reported. In the Zakrzewska-Pniewska et al. study [56], it was stated that dysautonomia was mild in 66% of cases, while in the Toru et al. study [35], only the prevalence of dysautonomia among groups was reported. Two studies [41,59] reported the prevalence of autonomic symptoms in various types of dementia: AD, DLB, VAD, PDD, and HC. Finally, in the retrospective study by Freidenberg et al. [48], the percentages of symptoms attributed to orthostatic hypotension (OH)—such as altered motor function (e.g., being found on the floor, head drop), diminished energy and wakefulness, dizziness, and confusion—were reported. Interestingly, in the Idiaquez et al. study [50], the authors stated that even though postprandial hypotension was observed in AD patients, no associated symptoms were reported.

### 3.2. Tools of Assessment

#### 3.2.1. HRV

HRV was the most popular tool, appearing in 12 out of 30 studies—either as the sole outcome measure or in combination with other autonomic tests, such as the SSR [34] and OH [29]. The RRIV test was used in combination with other autonomic tests (SSR and OH) in two studies [56,57]. A variation of the test, HRV related to body movement during sleep, was used in one study [47].

Among the HRV studies, one was observational and had no control group [42], and two studies compared patients with AD to DLB patients [34,37]. The other studies compared patients with AD with either a health control (HC) group or with multiple groups, including HC and patients with other types of dementia (VAD, DLB, PDD). In the two studies where patients with AD were compared only with patients with DLB [34,37], the findings were consistent. HRV was significantly lower in the DLB group compared to the AD group. In the three studies where AD was compared only with HC [38,45,46], the findings were also consistent: HRV was significantly lower in AD patients compared with controls.

There were six studies comparing more than two groups: three studies compared AD with HC and DLB [39,40,41], one study compared AD with HC and VAD [43], one compared AD with HC and MCI [44], and one included five groups (AD, HC, DLB, VAD, and PDD) [29]. Only the study [44] that compared patients with AD with patients with MCI and HC showed a significant difference in HRV between groups, and, more specifically, LF and HF power spectral mean values were significantly lower in patients with AD than in those with MCI and controls. In the remaining four studies, HRV in AD did not differ from HC, but it differed significantly from that in DLB patients. In the observational study by Toledo and Junqueira [42], the main finding was a positive correlation between cognitive performance and high-frequency power, and a negative correlation with the low-frequency spectrum.

RRIV, another test that measures heart rate variability, was used as a tool in two studies [56,57], and both reached the same conclusion: RRIV was significantly reduced in AD patients compared to HC. Lastly, the study that evaluated body movement-related HRV in AD and HC found that the ratio of the longest R-R interval before spontaneous body movements during sleep to the shortest was significantly lower in AD patients compared to controls. This ratio is an index of tonic heart rate modifications associated with abrupt sympathetic activation.

#### 3.2.2. OH

OH appeared in six studies as a tool to investigate autonomic dysfunction in AD. In three studies, OH was the only outcome measure [30,48,49]. In the study by Allan et al. [29], OH was studied along with HRV. In the study by Wang et al. [57], OH was studied along with RRIV and SSR, and in the study by Vitiello et al. [54], OH was studied along with norepinephrine (NE) measurements. One study was observational without HC [48]. In four studies, AD patients were compared to HC [30,49,54,57], and in the Allan et al. study [29], AD patients were compared with HC and three other patient groups. In the large chart review study by Freidenberg et al. [48], clinical symptoms and signs of OH were observed in 40% of AD patients and in 60% of patients suffering from non-Alzheimer dementia. Similarly, in Siennicki-Lantz et al. [30], a cross-sectional study, OH was observed in 4/12 (33%) of AD patients and in 9/15 (60%) HC. The two groups were not compared on the basis of OH occurrence, but on the basis of cerebral blood flow (CBF). AD patients with OH had lower CBF in frontal and parieto-frontal lobes compared to AD patients without OH. No such difference was observed on HC with and without OH. In the study by Wang et al. [57], changes in blood pressure and heart rate from the supine position to 3 min after standing did not differ significantly between AD patients and HCs. In two other studies [29,54], the mean drop in systolic blood pressure upon standing in AD patients differed significantly from that in HCs. In the study by Allan et al. [29], the mean change in systolic blood pressure during phase IV of the Valsalva maneuver and the mean change in diastolic blood pressure during isometric exercise showed differences between AD patients and the other patient groups (DLB, PDD). However, no significant differences were observed when AD patients were compared to HCs. In the study by Vitiello et al. [54], blood pressure decreased upon standing in AD patients and increased in HCs. The completely opposite finding was observed in the study by van Beek et al. [49], where blood pressure was higher in AD patients compared to controls after orthostasis.

#### 3.2.3. PPH

Postprandial hypotension was studied in AD patients in one paper [50], where patients were compared to HCs. A drop in systolic blood pressure was significantly more common in AD patients than in controls, even though the patients did not report any symptoms.

#### 3.2.4. SSR

SSR was used in combination with other autonomic tests (RRIV, TILT TEST, HRV, and OH) in four studies. In two studies [34,35], there was no control group. AD patients were compared to other patients suffering from DLB and/or PDD. In both cases, SSR abnormalities were more pronounced in DLB/PDD patients than in AD patients, and this difference reached statistical significance in the study by Toru et al. [35]. In contrast, in the study by Negami [34] et al., the SSR test showed greater sensitivity and specificity in detecting DLB than AD. The remaining two studies [56,57] used HC as a comparison group. Their findings were identical: SSR was normal in AD patients and did not differ from that in HCs. It is important to note that different SSR parameters were reported in each study. Negami et al. [34] measured only the amplitude from the palm, Zakrzewska-Pniewska et al. [56] measured only latency from both the palm and the sole, and Wang et al. [57]. measured both latency and amplitude from both the palm and the sole. Finally, Toru et al. [35] recorded SSR from both the palm and soles but reported only qualitative results (absence or low response).

#### 3.2.5. TILT TEST

The Tilt test was used in three studies, always in combination with another autonomic test (HRV, SSR, deep breathing). In Mellingsæter et al. [27], HRV indices differed significantly at 70° tilt in AD patients compared to HC. In the Elmståhl et al. [58] study, the cardiovascular responses (acceleration and brake indexes) to tilting differed significantly from HC. Both studies compared AD patients to HC. In the third study by Toru et al. [35], comparisons were made between AD and other patient groups (DLB, PDD). As mentioned earlier, results were given in a qualitative way. It is reported that changes in blood pressure in the Tilt test were significantly higher in DLB/PDD patients than in AD patients.

#### 3.2.6. 123. I-MIBG

^123^I-MIBG cardiac scintigraphy was performed in two studies [34,35] in combination with SSR. Neither of the two studies had a healthy control group; instead, they compared AD patients to DLB patients. In both cases, the heart-to-mediastinum ratio uptake (H/M) on the MIBG scan was significantly lower in DLB than in AD.

#### 3.2.7. Serum /CSF Norepinephrine

Norepinephrine concentrations were measured in five studies. In Pascualy et al. [52], NE was the only measurement tool to assess ANS function in AD. Basal NE concentrations were significantly higher in the AD group compared to HC. On the contrary, in the Vitiello et al. study [54], NE did not differ from controls, neither at baseline nor after standing. In two studies [53,55], NE in CSF and plasma were higher in advanced compared to moderate AD and HC. In the study by Toru et al. [35], AD patients were compared to DLB/PDD. With subjects in a supine position after at least 20 min of rest, NE values were significantly lower in DLB/PDD patients than in AD patients.

#### 3.2.8. Deep Breathing

In one study [58], the heart-rate expiration/inspiration ratio was measured in AD and HC patients and was found not to differ between the groups.

#### 3.2.9. BR

Baroreflex was used as the only tool to investigate dysautonomia in AD in two studies [32,51]. AD patients were compared to HCs and those with MCI/PD. In both studies, the findings were consistent: BR was reduced in AD compared to HCs. Finally, two papers [59,60] studied the prevalence of dysautonomic symptoms in AD using clinical scales and found no significant difference between AD and HCs.

## 4. Discussion

To the best of our knowledge, this is the first systematic review of autonomic dysfunction in AD. In 2011, Idiaquez and Roman [63] published a non-systematic review article on autonomic dysfunction in neurodegenerative dementias, including not only AD but all types of dementias. In 2014, Femminella et al. [16] provided a narrative review of the tools used to assess ANS dysfunction in AD and the related literature, though some studies were missing. Da Silva et al. [64], in 2018, and Cheng et al. [65], in 2020, each published a systematic review and meta-analysis of HRV findings in patients with dementia (not exclusively AD). There are also several review articles on non-cognitive symptoms in AD and other types of dementia [5,6,66,67,68], though they are not specifically dedicated to investigating dysautonomia in AD.

The main finding of the present review is that AD patients—even those who do not experience clinical dysautonomia—may show pathological results in certain tests or differ from age-matched healthy controls. However, these findings were not as pronounced as in the case of DLB, where dysautonomia is more clinically prominent and test results differ significantly not only from those of HCs but also from those of AD patients.

Based on these findings, certain questions arise regarding the involvement of the ANS in the course of AD. In this systematic review, most studies did not report any clinical signs of dysautonomia. In some cases, the prevalence of dysautonomia was similar to that in healthy elderly controls, or, in others, test results were pathological—as in the case of postprandial hypotension—yet patients reported no related symptoms during testing. On these grounds, one might question the clinical significance of this ‘laboratory-confirmed’ dysautonomia

Another interesting finding is that for the same laboratory procedure, the results varied across studies. HRV was the most commonly used autonomic test, but the design of the studies varied considerably. In all studies [38,45,46,47] where AD patients were compared only to healthy controls (HC), HRV was found to differ significantly between the groups, though no clinical symptoms were reported. When an additional patient group—such as those with VAD, DLB, or PDD—was included in the comparison, AD patients often did not differ from HCs, but showed significant differences only from those with DLB. This pattern was even more robust in studies where AD patients were compared exclusively to DLB patients [34,37]. Based on these findings, one can conclude that HRV is capable of distinguishing AD from DLB, but not consistently from HC.

Similarly inconclusive were the findings regarding the prevalence and presentation of orthostatic hypotension (OH) in AD and HC. In two studies, results were identical in AD and HC; in one study, OH was more prominent in AD, whereas, in another, unexpectedly, blood pressure was better preserved after standing in AD than in controls. Norepinephrine (NE) measurements were also conflicting, showing differences from HC in some studies and identical results in others. Interestingly, two studies reported that NE levels differed between advanced and moderate stages of AD, a point not addressed in other research. SSR is another example of a test that, while not able to differentiate between AD and HC, was capable of distinguishing AD from DLB.

Other tests showed more consistent results. Baroreflex (BR) and postprandial hypotension (PPH) differed between AD and HC, but patients did not report any autonomic symptoms, making the clinical significance of these findings uncertain. ^123^I-MIBG uptake was also consistently lower in AD compared to DLB, although no comparisons were made with HC. The tilt test showed consistent results across all studies in which it was used, effectively distinguishing AD from both HC and DLB patients.

Even more interesting is the fact that in studies where more than one autonomic test was applied, the results were not always consistent with one another. This could suggest that some tests may be more sensitive in detecting dysautonomia in this specific population. In this context of inconsistency, OH differed in AD patients in two studies, while HRV and NE levels appeared normal. In contrast, a third study showed the opposite pattern: OH did not differ between patients and controls, whereas another test—the RR interval variability (RRIV)—showed a significant difference. Therefore, the hypothesis that one test is more sensitive than another cannot be fully supported.

Among the studies that reported dysautonomia in AD, several hypotheses have been proposed to describe the underlying neuronal substrate. The general consensus is that the exact pathophysiology remains largely unknown. Most authors suggest that a cholinergic deficit disrupts the sympathetic/parasympathetic balance in AD, potentially contributing to dysautonomia [27,29,30,38,44,45,46,51,53,56,57,58]. This cholinergic depletion is thought to result from neurofibrillary tangles affecting the nucleus basalis of Meynert and from increased cholinesterase activity. On this basis, treatment with cholinesterase inhibitors has been shown to normalize some aspects of autonomic dysfunction in AD [45]. Other authors highlight the role of ‘autonomic-related cortices’ in AD pathophysiology, including the insular cortex, the ventromedial frontal (VMF) cortex, and the hypothalamus [30,32,51,57,58].

### Limitations

Several limitations of this study should be discussed. The majority of the included studies were cross-sectional studies. Despite the benefits offered by this type of research, such as the opportunity to establish preliminary evidence, determine the prevalence, and study the associations of multiple exposures and outcomes, there are obvious drawbacks. Cross-sectional studies are prone to recall and nonresponse bias, where people with the disease are more likely to remember and report symptoms or exposures than controls. Moreover, cross-sectional studies are unable to investigate the temporal relationship between outcomes and risk factors. Consequently, the results obtained from these studies are not capable of establishing causality. Instead, they can only establish associations, and sometimes, the associations identified might be difficult to interpret [69].

Another important limitation is the heterogeneity of the study populations. AD diagnoses were made clinically, based on scores from various cognitive tests, and in some cases supported by paraclinical findings—primarily imaging studies. In four studies [37,40,48,51], no diagnostic criteria were reported for recruiting AD patients. In the remaining studies, the most commonly used criteria were the NINCDS-ADRDA criteria [70] (National Institute of Neurological and Communicative Diseases and Stroke–Alzheimer’s Disease and Related Disorders Association), and, less commonly, the DSM (Diagnostic and Statistical Manual of Mental Disorders) editions III, IV, or V. Additionally, in most studies, no subgroup analyses by AD severity (mild, moderate, or severe) were reported, with the exception of the study by Raskind et al. [55], where AD patients were evaluated separately according to disease stage (moderate or advanced). It is also important to note that the control groups were not always composed of healthy individuals; in some cases, they included patients with other forms of dementia, which further complicates comparisons across studies.

Another significant limitation was the heterogeneity in outcome reporting. For example, different studies reported different HRV indices or measured different SSR parameters, making comparisons extremely challenging. Finally, the majority of studies did not report the presence or absence of dysautonomic symptoms, leaving it unclear whether such symptoms were not assessed or simply not detected. As a result, interpreting autonomic test findings in isolation is of uncertain clinical significance. Longitudinal studies are warranted to investigate, using refined clinical testing for ANS dysfunction, whether abnormalities in autonomic testing may precede clinical symptoms or serve as prognostic biomarkers.

## 5. Conclusions

AD is considered a systemic disease, with cognitive impairment as the primary manifestation. However, other areas beyond the neocortex also appear to be involved, leading to non-cognitive symptoms. The involvement of the ANS in AD has been demonstrated through various tests. Nonetheless, the findings of these tests have often been confusing or contradictory and less clear-cut compared to those observed in cases of DLB. DLB represents the second most prevalent cause of dementia after AD, and there is a possibility of shared pathologies, specifically amyloid plaques and neurofibrillary tangles, which complicates the process of differential diagnosis. In view of the above, the absence of clinically significant dysautonomia in AD, whilst the autonomic tests reveal abnormal results, may indicate that certain cases diagnosed as AD may in fact be DLB with concomitant AD pathology (DLB/AD+).

Therefore, despite the infrequency with which patients with AD report autonomic symptoms, it may be beneficial to systematically evaluate at least orthostatic hypotension (OH) and treat it accordingly. This is important because ANS involvement can lead to symptoms such as dizziness, syncope, and falls, which increase the risk of mortality in patients with AD. Given the inconsistencies and limitations discussed, establishing a clear and consistent picture of dysautonomia in AD based on the current literature remains challenging. The present findings underscore the need for further research in this field and highlight the importance of standardized clinical testing using validated assessment tools for ANS dysfunction. Such testing should ideally be incorporated into longitudinal study designs involving well-characterized patient populations. Thus, further research is required to enhance our understanding of the role of ANS in AD.

## Figures and Tables

**Table 1:** Demographic and clinical characteristics of the included studies.

No	Study	Sample Size	AGE Mean (SD)	Sex (F%)	MMSE GDS, FAB CAMCOG	Clinical Dysautonomia	Test	Findings	Comments
1	Haglund et al., 2024 [37]	18 AD 18 DLB	62.6 (6.2) 64.6 (9.3)	(61) (28.5)		5% OH	HRV	DLB < AD	Unknown clinical significance
2	Nair et al., 2023 [38]	20 AD 20 HC	Age matched	gender matched		No information	HRV	AD < HC LF/HF AD > HC	Sympathetic/parasympathetic balance disorder
3	Omoya et al., 2020 [39]	29 AD 26 DLB 25 HC	81.2 (6.4) 78.3 (8.1) 77.2 (5.4)	(65.5) (53.8) (68)	18.7 (4.7) 17.4 (7.2) 28.8 (1.0)	No information	HRV	LF DLB < AD late HRR (H_max_ − H_180_) AD < HC	AD exhibited differences from HC but not as large as in DLB
4	Kim et al., 2018 [40]	32 AD 23 DLB 36 HC	70.1 (6.3) 70.6 (5.2) 68.4 (6.4)	(56.3)	24.7 (2.8) 25.0 (2.4) 26.6 (1.9)	No information	HRV	AD = HC	HRV negatively correlated with verbal memory HRV can discriminate DLB-AD at the MCI stage
5	Kasanuki et al., 2015 [41]	30 AD 30 DLB 20 HC	79.8 (5.6) 79.9 (4.7) 77.2 (4.8)	(50) (46.6) (50)	20.5 (4.2) 18.0 (4.2) 30.0 (0.7)	Constipation, orthostatic dizziness, urinary incontinence, increased sweating	HRV	DLB < AD	HRV can differentiate DLB-AD
6	Toledo and Junqueira, 2010 [42]	22 AD	79.6 (1.4)	(90.9)		No information	HRV	HF-positive correlation, LF-negative correlation with cognitive performance	Severe cognitive deficiency →lower parasympathetic/higher cardiac sympathetic modulation.
7	Allan et al., 2005 [43]	14 AD 20 VAD 80 HC	77.2 (6.2) 81.4 (5.0) 75.7 (5.7)	(71.4) (33.3) (50)	18.4 (4.3) 18.6 (6.1) 28.5 (1.6)	No information	HRV	AD = VAD = HC	Insensitive to detect minor changes Sensitive for clinically significant dysautonomia
8	Zulli et al., 2005 [44]	33 AD 39 MCI 29 HC	72.1 (8.2) 70.0 (7.2) 69.8 (5.3)	(60.6) (58.9) (65.5)	19.0 (4.3) 27.4 (1.8) 28.8 (1.3)	No information	HRV	AD < MCI/HC	Correlated with the degree of cognitive impairment
9	Giubilei et al., 1998 [45]	12 AD 10 HC	65.7 (6.2)	(58.3)	18.4 (3.6)	No information	HRV	AD < HC	Treatment caused changes similar to those observed in controls
10	Aharon-Peretz et al., 1992 [46]	20 AD 7 HC	71.3 (8.2) 65 (2.4)		18.35 (3.18)	No information	HRV	AD < HC LF/HF AD > HC	Hypersympathetic, hypoparasympathetic state.
11	Allan et al., 2007 [29]	39 AD 29 VAD 27 DLB 37 PDD 38 HC	79 (6) 80 (6) 75 (7) 72 (5) 76 (7)	(56) (30) (43) (42) (53)		autonomic neuropathy 13%	HRV OH	→AD = HC →AD ≠ HC (prevalence)	Autonomic functions unimpaired except OH
12	Franceschi et al., 1986 [47]	16 AD 7 HC	62.2 (4.1) 59.6 (2.5)	(50) (42.8)		No information	HRV body movement related	Rmb AD < HC Rs/w AD = HC	Sympathetic cardiac dysfunction during sleep Unknown clinical importance
13	Freidenberg et al., 2013 [48]	188	80.8 (7.7)				OH	40% OH (prevalence)	Mental and motor fluctuations related to OH not traditional signs
14	van Beek et al., 2010 [49]	21 AD 20 HC	NO INF			No information	OH	AD > HC	Enhanced sympathetic tone Increased orthostatic tolerance
15	Siennicki-Lantz et al., 1999 [30]	12 AD 15 HC	82.6 (3.8) 81.8 (3.5)	(100) (100)		No information	OH	AD = HC 33% OH	AD with OH had significantly lower CBF frontal/parieto-frontal regions, compared with AD without OH
16	Idiaquez et al., 1997 [50]	10 AD 23 HC	73.9 (6.4) 71.5 (6.4)	(70) (47.8)	14.5 (5.85) 27.2 (1.6)	No associated symptoms	PPH	AD > HC	Sympathetic dysfunction— BP instability of central origin
17	Meel-van den Abeelen et al., 2012 [32]	18 AD 11 MCI 19 HC	72 (6) 74 (9) 75 (3)	(61.1) (54.5) (26.3)	22 (5) 25 (3) 29(5)	No information	BR	AD < HC	Not known significance
18	Szili-Török et al., 2001 [51]	24 AD 23 PD 22 HC	72.3 (7.2) 65 (9.3) 70 (6.6)	(60.8) (54.2) (68.2)	19 (6.5) 27.5 (2) 29 (0.9)	No information	BR	AD/PD < HC	Unclear significance in neurological disorders
19	Pascualy et al., 2000 [52]	9 AD 9 HC	76 (2) 76 (1)	(55.5) (55.5)	17 (1) 29 (1)	No information	Basal NE (CPT) responses to 1-min	Basal NE AD > HC NE (CPT) AD = HC	increased HPA axis responsiveness to CPT
20	Elrod et al., 1997 [53]	74 AD 42 HC 54 HC	69 (6) 68 (7) 26 (3)	(24.3) (38) (0)	18 (4)	No information	NE in CSF	Advanced AD > moderate AD/HC	Contribute to cognitive deficits and agitated behaviors
21	Vitiello et al., 1993 [54]	60 AD 20 HC	65.5 (8) 64.7 (9.4)	(53.3) (50)	4.6 (1.0) 0	No information	NE OH	→AD = HC →AD < HC (systolic BP)	More evident in depressed AD
22	Raskind et al., 1984 [55]	9 AD 7AD 6 HC	68 (8) 61 (6) 67 (10)	(0) (0) (0)	Advanced moderate	No information	NE in plasma/CSF	Advanced AD > moderate AD/HC	Contribute to agitation, sleep disturbance
23	Toru et al., 2018 [35]	59 AD 56 DLB 37 PDD	77.6 (6.9) 77.6 (6.5) 77.6 (6.6)	(62.7) (55) (59.4)	19.2 (5.7) 19.0 (9.3) 19.1 (7.2)	18.6% 100% 100%	SSR HUTiltTest NE ^123^I-MIBG	→AD ≠ DLB PDD →AD ≠ DLB PDD →AD > DLB/PDD →AD > DLB/PDD	SSR was the best marker of autonomic dysfunction for distinguishing DLB/PDD from AD
24	Negami et al., 2013 [34]	20 AD 20 DLB	78.5 (5.0) 78.7 (6.9)	(50) (50)	19.3 (3.6) 19.2 (4.8)	No information	SSR_amp_ HRV ^123^I-MIBG	→DLB < AD →DLB < AD →DLB < AD	SSR showed the most significant difference
25	Zakrzewska-Pniewska et al., 2012 [56]	54 AD 37 HC	73.1 (6.3) 47.3 (16.4)	(57.4) (56.7)		Mild in 66%	SSR RRIV	→AD = HC →AD < HC	Subclinical dysautonomia associated with cardiovascular dysfunction
26	Wang et al., 1994 [57]	23 AD 23 HC	70.7 (6.7) 70.8 (6.9)	(56.5) (39.1)	10.0 (6.9)	No information	SSR RRIV OH	→AD = HC →AD < HC →AD = HC	Mildly impaired vagal parasympathetic functions.
27	Mellingsæter et al., 2015 [27]	14 AD 48 HC	73.6 (5.7) 72 (6)	(50) (50)	24.8 (2.4)	No information	TILT	AD ≠ HC	Poorer sympathetic response to orthostatic stress
28	Elmståhl et al., 1992 [58]	24 AD 54 HC	85 (5.3)	(100)		No information	TILT TEST DEEP BREATHING	→AD ≠ HC →AD = HC	Imbalance of ANS Degeneration of cholinergic nuclei of Meynert
29	Allan et al., 2006 [59]	40 AD 46 PDD 32 DLB 38 VAD 42 HC	78 (5.6) 72 (5.7) 75 (7.1) 79 (5.9) 76 (6.7)	(55) (39) (41) (29) (48)	59 (14.5) 64 (16.3) 60 (15.0 62 (18.3) 94 (4.7)	Mucosal dryness urinary symptoms, constipation, postural dizziness	Autonomic symptom scale	AD = HC DLB PDD ≠ AD/HC	No clinically significant dysautonomia in AD
30	Allan et al., 2009 [60]	38 AD 40 PDD 30 DLB 32 VAD 39 HC	79 (5.8) 72 (6.0) 76 (7.1) 79 (6.2) 75 (6.4)	(52.6) (35) (40) (27.1) (46.2)	59 (15) 64 (16) 59 (15) 64 (18) 94 (4.7)	Symptomatic orthostatic hypotension dizziness, lightheadedness, unsteadiness or presyncope Ewing’s battery	Ewing’s battery Falls incidence	AD/VAD = HC	Higher incidence of falls in all dementia types than HC. PDD/DLB at higher risk

AD: Alzheimer’s disease, BP: blood pressure, BR: baroreflex, CBF: cerebral blood flow, CPT: cold pressor test, CSF: cerebrospinal fluid, DLB: dementia with Lewy bodies, FAB: frontal assessment battery, HF: high frequency, HPA: hypothalamic-pituitary-adrenocortical axis, HRR: heart rate recovery, HUTiltTest: Head-Up Tilt Test, LF: low frequency, MCI: mild cognitive impairment, ^123^I-MIBG: ^123^I-Meta-Iodobenzylguanidine Cardiac Scintigraphy, MMSE: Mini Mental State Examination, NE: norepinephrine, OH: orthostatic hypotension, PDD: Parkinson’s disease dementia, PPH: postprandial hypotension, RRIV: R-R interval variation test, Rmb: related to body movement, Rs/w: sleep wakefulness ratio, VAD: vascular dementia. Symbol explanation: ‘=’ indicates no difference between groups; ‘<’ or ‘>’ indicates which group showed larger or smaller values in the specific test; ‘→’ was used in case of multiple test application to indicate which test the equation applies to.

## Supplementary Materials

The following supporting information can be downloaded at: https://www.mdpi.com/article/10.3390/brainsci15050502/s1, File S1: DETAILS OF THE SEARCH; File S2: EXCLUDED REPORTS; File S3: JBI CRITICAL APPRAISAL CHECKLIST FOR ANALYTICAL CROSS SECTIONAL STUDIES. References [71,72,73,74,75,76,77,78,79,80,81,82,83,84] are cited in the supplementary materials.

## Abbreviations

The following abbreviations are used in this manuscript:

DSM	American Psychiatric Association’s Diagnostic and Statistical Manual
AD	Alzheimer’s disease
PD	Parkinson’s disease
COMPASS	Composite Autonomic Symptom Score
HRV	heart rate variability
LF	low frequency
HF	high frequency
VLF	very low frequency
TSP	total spectral power
ANS	autonomic nervous system
BPV	blood pressure variability
RRIV	R-R interval variation
ECG	electrocardiogram
OH	orthostatic hypotension
PPH	postprandial hypotension
BR	baroreflex
SSR	Sympathetic Skin Response
PRISMA	Preferred Reporting Items for Systematic Reviews and Meta-Analyses
HC	healthy controls
VAD	vascular dementia
MCI	mild cognitive impairment
DLB	dementia with Lewy bodies
PDD	Parkinson’s disease dementia
MMSE	Mini Mental State Examination
CAMCOG	Cambridge Cognition Examination
GDS	Global Deterioration Scale
FAB	frontal assessment battery
NE	norepinephrine
