# Supplementary material for: Dysautonomia in Alzheimer’s Disease: A Systematic Review
Source: Brain Sci. 2025 May 14;15(5):502. doi: 10.3390/brainsci15050502 (PMC12109965; doi:10.3390/brainsci15050502)
Supplement: Supplementary file 1 [file brainsci-15-00502-s001.zip › brainsci-3640081-supplementary1.pdf]

SUPPLEMENT 1

("Alzheimer's disease"[MeSH Terms] OR ("Alzheimer"[All Fields] AND "disease"[All Fields]) OR "Alzheimer's disease"[All Fields] OR ("Alzheimer's"[All Fields] AND "disease"[All Fields]) OR "Alzheimer's disease"[All Fields]) AND ("autonomic nervous system diseases"[MeSH Terms] OR ("autonomic"[All Fields] AND "nervous"[All Fields] AND "system"[All Fields] AND "diseases"[All Fields]) OR "autonomic nervous system diseases"[All Fields] OR "dysautonomia"[All Fields] OR "dysautonomias"[All Fields])

"Alzheimer's disease"[MeSH Terms] OR ("Alzheimer"[All Fields] AND "disease"[All Fields]) OR "Alzheimer's disease"[All Fields] OR ("Alzheimer's"[All Fields] AND "disease"[All Fields]) OR "Alzheimer's disease"[All Fields]) AND (("heart rate"[MeSH Terms] OR ("heart"[All Fields] AND "rate"[All Fields]) OR "heart rate"[All Fields]) AND ("variabilities"[All Fields] OR "variability"[All Fields] OR "variable"[All Fields] OR "variable s"[All Fields] OR "variables"[All Fields] OR "variably"[All Fields]))

("Alzheimer's disease"[MeSH Terms] OR ("Alzheimer"[All Fields] AND "disease"[All Fields]) OR "Alzheimer's disease"[All Fields] OR ("Alzheimer's"[All Fields] AND "disease"[All Fields]) OR "Alzheimer's disease"[All Fields]) AND (("sympathetic"[All Fields] OR "sympathetically"[All Fields] OR "sympathetics"[All Fields]) AND ("skin"[MeSH Terms] OR "skin"[All Fields]) AND ("response"[All Fields] OR "responses"[All Fields] OR "responsive"[All Fields] OR "responsiveness"[All Fields] OR "responsivenesses"[All Fields] OR "responsives"[All Fields] OR "responsivities"[All Fields] OR "responsivity"[All Fields]))

("Alzheimer's disease"[MeSH Terms] OR ("Alzheimer"[All Fields] AND "disease"[All Fields]) OR "Alzheimer's disease"[All Fields] OR ("Alzheimer's"[All Fields] AND "disease"[All Fields]) OR "Alzheimer's disease"[All Fields]) AND ("hypotension, orthostatic"[MeSH Terms] OR ("hypotension"[All Fields] AND "orthostatic"[All Fields]) OR "orthostatic hypotension"[All Fields] OR ("orthostatic"[All Fields] AND "hypotension"[All Fields]))
