# Supplementary material for: Dysautonomia in Alzheimer’s Disease: A Systematic Review
Source: Brain Sci. 2025 May 14;15(5):502. doi: 10.3390/brainsci15050502 (PMC12109965; doi:10.3390/brainsci15050502)
Supplement: Supplementary file 1 [file brainsci-15-00502-s001.zip › brainsci-3640081-supplementary2.pdf]

SUPPLEMENT 2 EXCLUDED REPORTS

Not in English language (n=1)

Paradowski B, Bilińska M, Koszewicz M, Pokryszko A. Ocena czynności układu sercowo-naczyniowego i potowydzielniczego u chorych na chorobe Alzheimera [Evaluation of cardiovascular and sudomotor functions in Alzheimer's disease]. Pol Merkur Lekarski. 1999 Oct;7(40):180-4. Polish. PMID: 10835909.Review (n=6)

Review (n=6)

Tulbă D, Cozma L, Popescu BO, Davidescu EI. Dysautonomia in Alzheimer's Disease. Medicina (Kaunas). 2020 Jul 8;56(7):337. doi: 10.3390/medicina56070337. PMID: 32650427; PMCID: PMC7404689.

Elia A, Fossati S. Autonomic nervous system and cardiac neuro-signaling pathway modulation in cardiovascular disorders and Alzheimer's disease. Front Physiol. 2023 Jan 30;14:1060666. doi: 10.3389/fphys.2023.1060666. PMID: 36798942; PMCID: PMC9926972.

Idiaquez J, Roman GC. Autonomic dysfunction in neurodegenerative dementias. J Neurol Sci. 2011 Jun 15;305(1-2):22-7. doi: 10.1016/j.jns.2011.02.033. Epub 2011 Mar 25. PMID: 21440258.

Kenny RA, Kalaria R, Ballard C. Neurocardiovascular instability in cognitive impairment and dementia. Ann N Y Acad Sci. 2002 Nov;977:183-95. doi: 10.1111/j.1749-6632.2002.tb04816.x. PMID: 12480751.

Mendelsohn AR, Larrick JW. The Danger of Being Too Sympathetic: Norepinephrine in Alzheimer's Disease and Graying of Hair. Rejuvenation Res. 2020 Feb;23(1):68-72. doi: 10.1089/rej.2020.2309. Epub 2020 Feb 11. PMID: 31989871.

Royall DR, Gao JH, Kellogg DL Jr. Insular Alzheimer's disease pathology as a cause of "age-related" autonomic dysfunction and mortality in the non-demented elderly. Med Hypotheses. 2006;67(4):747-58. doi: 10.1016/j.mehy.2005.10.036. Epub 2006 Jun 27. PMID: 16806725.

Not an AD diagnosis (n=3)

Nashiro K, Yoo HJ, Cho C, Kim AJ, Nasseri P, Min J, Dahl MJ, Mercer N, Choupan J, Choi P, Lee HRJ, Choi D, Alemu K, Herrera AY, Ng NF, Thayer JF, Mather M. Heart rate and breathing effects on attention and memory (HeartBEAM): study protocol for a randomized controlled trial in older adults. Trials. 2024 Mar 15;25(1):190. doi: 10.1186/s13063-024-07943-y. PMID: 38491546; PMCID: PMC10941428.

Liu CR, Yang CY, Sharma D, Chen TH, Huang XQ, Hung TM, Kuo TBJ, Jou JH. Associations between Sleep Duration and Autonomic Nervous System Regulation in Patients with Probable Alzheimer's Disease: A Cross-Sectional Pilot Study. Clocks Sleep. 2024 Sep 24;6(4):533-545. doi: 10.3390/clockssleep6040035. PMID: 39449309; PMCID: PMC11503315.

Gan J, Liu S, Wang XD, Hu W, Lv Y, Niu J, Meng X, Chen Y, Shi Z, Ji Y. The Association Between Hyperhidrosis and Dementia: A Community-Based Research. J Alzheimers Dis. 2021;84(4):1657-1667. doi: 10.3233/JAD-210611. PMID: 34744079.

Autopsy or non-clinical study (n=2)

Lin F, Ren P, Wang X, Anthony M, Tadin D, Heffner KL. Cortical thickness is associated with altered autonomic function in cognitively impaired and non-impaired older adults. J Physiol. 2017 Nov 15;595(22):6969-6978. doi: 10.1113/JP274714. Epub 2017 Oct 25. PMID: 28952161; PMCID: PMC5685832.

Fotiou DF, Stergiou V, Tsiptsios D, Lithari C, Nakou M, Karlovasitou A. Cholinergic deficiency in Alzheimer's and Parkinson's disease: evaluation with pupillometry. Int J Psychophysiol. 2009 Aug;73(2):143-9. doi: 10.1016/j.ijpsycho.2009.01.011. Epub 2009 May 3. PMID: 19414041.

ANS assessment was not the primary outcome (n=3)

Joshi, A., Mendez, M. F., Kaiser, N., Jimenez, E., Mather, M., & Shapira, J. S. (2014). Skin conductance levels may reflect emotional blunting in behavioral variant frontotemporal dementia. The Journal of Neuropsychiatry and Clinical Neurosciences, 26(3), 227–232. https://doi.org/10.1176/appi.neuropsych.12110332

Deutsch CK, Patnaik PP, Greco FA. Is There a Characteristic Autonomic Response During Outbursts of Combative Behavior in Dementia Patients? J Alzheimers Dis Rep. 2021 May 4;5(1):389-394. doi: 10.3233/ADR-210007. PMID: 34189410; PMCID: PMC8203282.

Mendez MF, Fong SS, Ashla MM, Jimenez EE, Carr AR. Skin Conduction Levels Differentiate Frontotemporal Dementia From Alzheimer's Disease. J Neuropsychiatry Clin Neurosci. 2018 Summer;30(3):208-213. doi: 10.1176/appi.neuropsych.17080168. Epub 2018 Apr 6. PMID: 29621927; PMCID: PMC6081247.
