# Supplementary material for: Dysautonomia in Alzheimer’s Disease: A Systematic Review
Source: Brain Sci. 2025 May 14;15(5):502. doi: 10.3390/brainsci15050502 (PMC12109965; doi:10.3390/brainsci15050502)
Supplement: Supplementary file 1 [file brainsci-15-00502-s001.zip › brainsci-3640081-supplementary3.pdf]

JBI CRITICAL APPRAISAL CHECKLIST FOR ANALYTICAL CROSS-SECTIONAL STUDIES

Study	Were the criteria for inclusion in the sample clearly defined?	Were the study subjects and the setting described in detail?	Was the exposure measured in a valid and reliable way?	Were objective, standard criteria used for measurement of the condition?	Were confounding factors identified?	Were strategies to deal with confounding factors stated?	Were the outcomes measured in a valid and reliable way?	Was appropriate statistical analysis used?	Overall appraisal
1	U	Y	Y	U	Y	Y	Y	Y	I
2	Y	Y	Y	Y	Y	Y	Y	Y	I
3	Y	Y	Y	Y	Y	Y	Y	Y	I
4	Y	Y	Y	U	Y	Y	Y	Y	I
5	Y	Y	Y	Y	Y	Y	Y	Y	I
7	Y	Y	Y	Y	Y	Y	Y	Y	I
8	Y	Y	Y	Y	Y	Y	Y	Y	I
9	U	Y	Y	Y	Y	Y	Y	Y	I
10	U	Y	Y	Y	U	U	Y	Y	I
11	Y	Y	Y	Y	Y	Y	Y	Y	I
12	U	Y	Y	U	Y	Y	Y	Y	I
14	U	Y	Y	Y	U	U	Y	Y	I
15	Y	Y	Y	Y	Y	Y	Y	Y	I
16	Y	Y	Y	Y	Y	Y	Y	Y	I
17	U	Y	Y	Y	Y	Y	Y	Y	I
18	Y	Y	Y	U	Y	Y	Y	Y	I
19	Y	Y	Y	Y	Y	Y	Y	Y	I
20	U	Y	Y	Y	Y	Y	Y	Y	I
21	Y	Y	Y	Y	Y	Y	Y	Y	I
22	Y	Y	Y	Y	Y	Y	Y	Y	I
23	Y	Y	Y	Y	Y	Y	Y	Y	I
24	U	Y	Y	Y	Y	Y	Y	Y	I
25	Y	Y	Y	Y	Y	Y	Y	Y	I
26	U	Y	Y	Y	Y	Y	Y	Y	I
27	Y	Y	Y	U	Y	Y	Y	Y	I
28	Y	Y	Y	Y	Y	Y	Y	Y	I
29	Y	Y	Y	Y	Y	Y	Y	Y	I
30	Y	Y	Y	Y	Y	Y	Y	Y	I

Y: Yes, N: No, U: Unclear, NA: Not applicable, I: Include, E: Exclude, S: Seek further info
